# Persistence of antibodies in laboratory staff immunized with quadrivalent meningococcal polysaccharide vaccine

**DOI:** 10.1186/1745-6673-8-4

**Published:** 2013-03-04

**Authors:** Johannes Elias, Jamie Findlow, Ray Borrow, Angelika Tremmel, Matthias Frosch, Ulrich Vogel

**Affiliations:** 1 Institute for Hygiene and Microbiology, University of Wuerzburg, Josef Schneider-Strasse 2, 97080, Wuerzburg, Germany; 2 Vaccine Evaluation Unit, Health Protection Agency, Manchester Medical Microbiology Partnership, P.O. Box 209, Clinical Sciences Building 2, Manchester Royal Infirmary, M13 9WZ, Manchester, UK

**Keywords:** Vaccination, Meningococcal infections, Biohazards, Meningococcal polysaccharide caccine

## Abstract

**Background:**

Occupational exposure to live meningococci can potentially cause invasive meningococcal disease in laboratory staff. While, until recently, immunization with quadrivalent polysaccharide vaccine represented one cornerstone of protection, data on long-term persistence of antibodies in adults remain scarce.

**Methods:**

We analyzed the relationship of antibody levels and time following quadrivalent polysaccharide vaccination (Mencevax® ACWY, GlaxoSmithKline) in a cross-sectional sample of 20 laboratory workers vaccinated at ages between 16.4 to 40.7 years from Germany. Sera were obtained 0.4 to 158.5 (median 35.3) months after vaccination. At the time of sampling, laboratory workers had been regularly exposed to meningococci for periods between 3.2 to 163.8 (median 41.2) months. Serum bactericidal assay (SBA) with rabbit complement and a microsphere-based flow analysis method were used to determine bactericidal titers and concentrations of IgG, respectively, against serogroups A, C, W135, and Y. Decay of antibodies was modeled using linear regression. Protective levels were defined as SBA titers ≥ 8.

**Results:**

Half-lives of SBA titers against serogroups A, C, W135, and Y were estimated at 27.4, 21.9, 18.8, and 28.0 months, respectively. Average durations of protection were estimated at 183.9, 182.0, 114.6, and 216.4 months, respectively. Inter-individual variation was high; using lower margins of 95% prediction intervals, minimal durations of protection against serogroups A, C, W135 and Y were estimated at 33.5, 24.6, 0.0, and 55.1 months, respectively. The proportion of staff with protective SBA titers against W135 (65.0%) was significantly lower than proportions protected against A (95.0%), C (94.7%), and Y (95.0%). Consistently, geometric mean titer (97.0) and geometric mean concentration of IgG (2.1 μg/ml) was lowest against serogroup W135. SBA titers in a subset of individuals with incomplete protection rose to ≥ 128 (≥ 8 fold) after reimmunization with a quadrivalent glycoconjugate vaccine.

**Conclusions:**

The average duration of protection following immunization with a quadrivalent polysaccharide vaccine in adults was ≥ 115 months regardless of serogroup. A substantial proportion (approximately 23% according to our decay model) of adult vaccinees may not retain protection against serogroup W135 for five years, the time suggested for reimmunization.

## Background

*Neisseria meningitidis*, commonly known as the meningococcus, is a Gram-negative bacterium that colonizes the pharyngeal mucosa of approximately 10% of humans in an age-dependent manner [1]. In rare cases, it causes potentially deadly invasive disease in the form of meningitis or sepsis in previously healthy individuals. Most cases appear in infants and adolescents [2], but adults over the age of 25 are also affected. According to their polysaccharide capsule, meningococci can be distinguished into twelve serogroups [3]. Globally, most cases of invasive disease are caused by serogroups A, B, C, W135, X, and Y. To date, licensed vaccines consisting of polysaccharide or glycoconjugate-formulations exist for the prevention of disease by serogroups A, C, W135, and Y.

Laboratory staff working with live cultures of meningococci are at a potentially increased risk of contracting disease. A study based on cases from the USA contrasted an attack rate of 13/100,000 among microbiologists between 1996 and 2001 to the incidence rate of 0.2/100,000 among adults in general [4]. Similarly, a UK analysis claimed that laboratory workers had a 184-fold increased risk of disease compared with the background population [5]. As lethality in documented laboratory-acquired cases reaches 50%, prevention to exposure of droplets or aerosols containing *N. meningitidis* by the use of biological safety cabinets is critical [4]. A further control measure is vaccination of staff, as 44% of cases are preventable by presently licensed vaccines [4]. Even though the recent death of a laboratory worker sadly underscores the current lack of vaccine preventability against serogroup B [6], the continuing importance of adequate immunization is emphasized by several reports of vaccine-preventable cases [7-9] that were published after the survey by Sejvar et al. [4].

Currently authorities in several countries including the Centers of Disease Control and Prevention (CDC), USA, Department of Health (DoH), UK, and Robert-Koch-Institute (RKI), Germany, recommend primary immunization of laboratory workers exposed to meningococci with quadrivalent glycoconjugate vaccine [10-12]. Nevertheless, a presumably large proportion of presently employed personnel received primary immunization with until recently recommended polysaccharide vaccine. DoH [10] and CDC [13] recommend revaccination of adults who had received a polysaccharide vaccine, if at continued risk, after five years. To our knowledge, recommendations regarding travelers do not differ regarding periods of reimmunization.

Serum-bactericidal antibody (SBA) titers of ≥ 8 using rabbit complement are regarded as the most practical correlate for protection against serogroup C disease and have since been applied to serogroup A, Y and W135 [14]. Levels of immunoglobulin, specifically concentrations exceeding 2 μg/ml, were only found useful for the prediction of protection against serogroup A [15]. It is the persistence of SBA after vaccination that warrants protection against invasive disease rather than immune memory, given that the rise in SBA after boosting is too slow to prevent invasive disease [16,17]. While soon after introduction of polysaccharide vaccines several reports have detailed the rapid decline of antibody levels after immunization in children [18,19], the length of persistence in adults has not been studied extensively. To our knowledge, only one report has investigated the duration of antibody response after immunization of adults with quadrivalent meningococcal polysaccharide targeting serogroups A, C, W135, and Y, suggesting persistence of bactericidal antibodies against serogroup C for up to ten years after vaccination [20]. Nevertheless, bactericidal activity was not followed up in that study for serogroups A, W135, and Y.

Our report documents antibody response directed against A, C, W135, and Y in a cross-sectional sample of sera obtained from 20 laboratory workers previously vaccinated once with a quadrivalent polysaccharide vaccine. The aim of our analysis is to describe and model the relationship of antibody levels and time passed since immunization.

## Methods

### Sera

A cross-sectional sample of sera from 20 laboratory staff employed at the Institute for Hygiene and Microbiology, University of Wuerzburg, Germany, was analyzed. Sera were collected between June 2008 and December 2010 and frozen at −80°C until analysis. Staff were eligible to participate if they had occupational exposure to live cultures of *Neisseria meningitidis* in the year before sampling and had been vaccinated once with a polysaccharide vaccine by the occupational health physician. Of 20 eligible laboratory workers all agreed to participate. Participants were informed that the analysis was part of an internal quality control in occupational health with the aim to identify individuals not possessing protective SBA titers. All participants had been immunized only once with Mencevax® ACWY (GlaxoSmithKline GmbH & Co., Munich, Germany), a meningococcal vaccine containing 50 μg each of serogroup A, C, W135, and Y capsular polysaccharide. The exact dates of vaccination were verified by examining the vaccination booklets of the participants. Serum samples were obtained 0.4 to 158.5 (median 35.3) months after vaccination and age of employees at vaccination ranged from 16.4 to 40.7 (median 25.6) years (Additional file 1: Table S1). Twelve of 20 (60%) participants were female. As part of their laboratory occupation employees were regularly exposed to live *N. meningitidis* for time periods between 3.2 to 163.8 months (median 41.2, interquartile range 28.7 to 74.1) before donating serum. SBA titers and IgG concentration against serogroup C was excluded for one individual (participant no. 17, Additional file 1: Table S1), who was additionally vaccinated with NeisVac-C® (Baxter Deutschland GmbH, Unterschleißheim, Germany), a glycoconjugate vaccine against serogroup C, six months after receiving polysaccharide vaccination. All participants received written statements regarding their level of protection. In addition, all participants signed written consents agreeing to the publication of their data in anonymized form in accordance with the Ethics Committee of the University of Wuerzburg.

### Serum bactericidal antibody activity

SBA was assessed with baby rabbit complement (Pel-Freez® Biologicals, AR, USA) using the method previously described [21], except that after incubation with serum, bacterial suspensions were dropped and tilted onto sheep blood plates (bioMérieux, Nürtingen, Germany) and grown overnight at 35°C and 5% CO_2_. The reference strains for serogroups A, C, W135, and Y were F8238, C11, M01.240070, and M00.242975, respectively. Colonies were counted with the ProtoCOL device (Synbiosis, Cambridge, UK). SBA titers were expressed as the reciprocal of the final serum dilution giving ≥ 50% killing at 60 minutes. Titers ≥ 8 were regarded as protective [14]. Titers < 4 were assigned a value of 2.

### Determination of IgG concentrations

Concentrations of IgG directed against capsular polysaccharide of serogroups A, C, W135, and Y were determined using a microsphere-based flow analysis method [22]. Sera were titrated against the International meningococcal standard reference serum CDC 1992 [23].

### Statistical analyses

To analyze the relationship between SBA titers or IgG concentrations and time after vaccination the following simplifying assumptions were used: 1) antibodies were expected to decay following a simple exponential model, described by *L* = *L*_0_ · *e*^*km*^, where *L* represents antibody level *m* months after attaining *L*_0_, *L*_0_ is the plateau level after vaccination, and *k* stands for the first-order rate constant; 2) we premised that plateau concentrations (*L*_0_) were attained 10 days after vaccination in each individual, as described in a Dutch study detailing daily changes in antibody levels in adults after primary immunization [16], after which decline according to above function set in. The value of *L*_0_ and *k* were estimated using linear regression of log-transformed titers or concentrations according to log(*L*) = *a*+ *km*, where *e*^*a*^ equals *L*_0_ and *k* can be conveniently extracted from the slope of the fitted line. Also, we assumed log-normal distribution of antibody levels. We chose months as temporal units, whereby one month was equivalent to 30.42 days. Half-life *λ* in months was calculated as $\lambda =\frac{\log\left(0.5\right)}{k}$. Differences in geometric mean titers (GMT) or concentrations (GMC) between serogroups were assessed using within-subjects ANOVA of log-transformed levels. The Friedman test, a nonparametric analogue, was additionally completed, if log-transformed data failed the Shapiro-Wilk test for normality (i.e. p < 0.05). Differences in the proportions of individuals with protective SBA titers (≥ 8), or IgG concentrations ≥ 2 μg/ml against the analyzed serogroups were evaluated with Cochran’s Q test. We used Pearson’s product moment correlation coefficient to describe association between log-transformed IgG concentrations and SBA titers. Linear regression was performed with the program R [24], version 2.14.1. Prediction intervals for fitted log-transformed antibody levels were generated with R’s built-in function “predict.lm”; percentiles of prediction intervals were computed assuming a symmetric distribution of forecast antibody levels centered on the value fitted by linear regression (equal to 50th percentile).

## Results

### Serum bactericidal antibody titers

SBA GMTs differed significantly across serogroups, as shown by within-subjects ANOVA of log-transformed values (p = 0.006; Table 1). The proportion of individuals within our sample presumably protected against disease, as inferred by the rate of sera with titers ≥ 8, differed significantly across serogroups (Cochran’s Q: 13.2 on 3 degrees of freedom, p = 0.004); protection against serogroup W135 was lowest with 65.0%, while protection exceeded 94.7% for all other serogroups (Table 1).

**Table 1:** Geometric means of antibody levels against serogroups A, C, W135, and Y

	**A**	**C**	**W135**	**Y**
SBA GMT	256.0	598.8	97.0	531.0
≥ 8 (%)	19 (95.0%)	18 (94.7%)	13 (65.0%)	19 (95.0%)
IgG GMC (μg/ml)	17.4	4.9	2.1	2.4
≥ 2 μg/ml (%)	20 (100.0%)	13 (68.4%)	8 (40.0%)	8 (40.0%)

*SBA*: serum bactericidal assay; *IgG*: Immunoglobulin G; *GMT*: geometric mean titer; *GMC*: geometric mean concentration in μg/ml.

### Decay models

**Figure 1:**
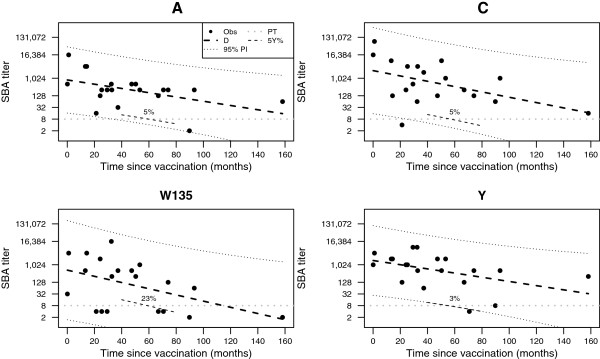
**Kinetics of serum bactericidal antibody titers against meningococci of serogroups A, C, W135, and Y.** Obs: observed titers, D: mean decay, PI: 95% prediction interval, PT: protective threshold, 5Y%: cropped contour line representing percentile of prediction interval below protective threshold five years after vaccination.

SBA titers against all serogroups significantly decreased with time (Figure 1), evidenced by negative slopes in linear regression (Table 2). Log-transformed SBA titers and residuals of linear models showed no evidence against normal distribution. W135 SBA titers showed the lowest estimated initial value (L_0_ = 541.5) and strongest decay (k = −0.037); nevertheless, estimated parameters for other serogroups (A, C, and Y) were within 95% confidence intervals of W135’s L_0_ (73.0 to 4,016.4) and k (−0.070 to – 0.003), and therefore not significantly different (Table 2). By extrapolation of the fitted line, average SBA titers for serogroups A, C, W135, and Y were expected to fall below the protective threshold of 8 at 183.9, 182.0, 114.6, and 216.4 months, respectively, after attaining L_0_. To account for high inter-individual variation, we used the crossing points of the lower margins of 95% prediction intervals with horizontal lines at the putative protective SBA titre of ≥ 8 to estimate probable minimal durations of protection (Figure 1): except for W135 (0.0 months), values surpassed 24 months after reaching L_0_ (A: 33.5, C: 24.6, Y: 55.1). Population estimates of the proportion of adults not protected after 5 years, the period suggested for reimmunization [10,13], were computed using the percentile of the models’ prediction intervals at 60 months intersecting the protection level: intervals equal or below the 5th, 5th, 23rd, and 3rd percentile corresponded to titers < 8 for serogroups A, C, W135, and Y, respectively (Figure 1).

**Table 2:** Estimated parameters of the decay of serum-bactericidal antibody titers after meningococcal polysaccharide vaccination

**Serogroup**	**L**_**0 **_**(CI)**	**k (CI)**	**λ (CI)**
A	834.1 (219; 3,176.5)	−0.025 (−0.048; -0.003)	27.4 (14.5; 248.9)
C	2,522.2 (442.2; 14,387.8)	−0.032 (−0.061; -0.002)	21.9 (11.3; 343.7)
W135	541.5 (73.0; 4,016.4)	−0.037 (−0.070; -0.003)	18.8 (9.8; 224.1)
Y	1,687 (413.3; 6,886.5)	−0.025 (−0.048; -0.001)	28.0 (14.3; 641.6)

*L*_*0*_: titer after vaccination, CI: 95% confidence interval, k: first-order rate constant, λ: half-life in months (derived from k).

### Concentration of IgG

**Figure 2:**
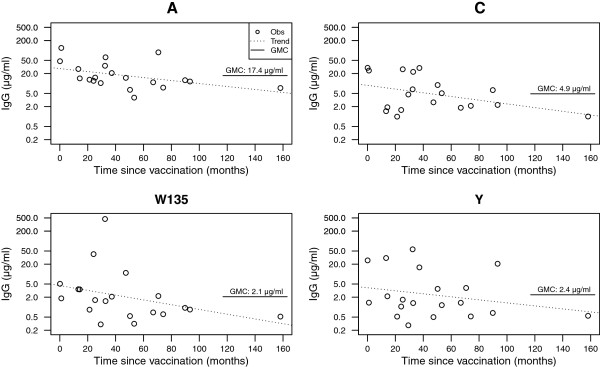
**Kinetics of serum IgG concentrations against capsular polysaccharides of meningococcal serogroups A, C, W135, and Y*.*** Obs: observed concentration; Trend: fitted line of linear models (slopes were not significantly different from zero for any of the serogroups); GMC: geometric mean concentration.

Linear regression of log-transformed concentration by time (months) predicted negative slopes for all serogroups (A: -0.010, C: -0.013, W135: -0.017, Y: -0.011), suggesting slight decay with time (Figure 2). Nevertheless, slopes were not significantly different from zero for any serogroup (Figure 2; A: p = 0.056, C: p = 0.081, W135: p = 0.122, and Y: p = 0.289). Consistent with SBA titers, however, both the GMC and fitted slope was lowest for W135 (2.1 μg/ml and −0.017, respectively). IgG GMCs differed significantly between serogroups according to within-subjects ANOVA of log-transformed values (p < 0.001). As log-transformed IgG concentrations against C, W135, and Y showed evidence against normal distribution, the Friedman test was additionally performed, which confirmed non-random distribution of concentrations across serogroups (p < 0.001). This was not an effect of the low concentration against W135 (2.1 μg/ml), which was close to C (4.9 μg/ml) and Y (2.4 μg/ml), but due to the high GMC of antibodies directed against A (17.4 μg/ml). Similarly, the significant difference in the proportion of individuals with concentrations equal or above 2 μg/ml (Cochran’s Q: 21.2, 3 d.f., p < 0.001) was a result of the high concentration of IgG against A: thus, 20 of 20 employees with regard to A, yet only 13, 8, and 8 with regard to C, W135, and Y, respectively, had concentrations surpassing 2 μg/ml (Table 1).

### Correlation of serum bactericidal antibody titers and IgG concentrations

A significant correlation of log-transformed SBA titers and IgG concentrations was demonstrated for serogroups C and W135, with coefficients of 0.670 (95% CI 0.356 to 0.874) and 0.581 (95% CI 0.186 to 0.814), respectively. In contrast, there was no conclusive evidence for association between SBA titers and IgG concentrations directed against serogroup A or Y with correlation coefficients of 0.382 (95% CI −0.073 to 0.705) and 0.094 (95% CI −0.364 to 0.515), respectively. Of note, the value for Y was barely above zero and significantly lower than estimates for C and W135.

### Serum bactericidal antibody titers after immunization with conjugate vaccine

Six of 20 participants (30%) showed insufficient protection against at least one of the serogroups tested (Additional file 1: Table S1) and were therefore offered vaccination with a quadrivalent glycoconjugate vaccine (Menveo®, Novartis Vaccines & Diagnostics GmbH, Marburg, Germany). Of four staff continuing to be employed at the research facility in Wuerzburg, three agreed to be revaccinated. Sera of these individuals were obtained four weeks after vaccination and assayed for SBA: all revaccinated individuals developed titers ≥ 128, corresponding to at least 8-fold increases compared to pre-boost values, against A, C, W135, and Y (Table 3).

**Table 3:** Serum-bactericidal antibody titers four weeks after application of a booster dose with a quadrivalent conjugate vaccine

**PN**	**Serum bactericidal antibody titers**			
	**A**	**C**	**W135**	**Y**
17	4,096 (16)	8,192 (−)	128 (32)	4,096 (1,024)
18	512 (256)	512 (8)	8,192 (4,096)	8,192 (1,024)
19	8,192 (128)	2,048 (128)	2,048 (1,024)	16,384 (64)

Titers are from three laboratory workers with low antibody titers. Parentheses contain fold increases compared to pre-boost titers (see Additional file 1: Table S1). *PN*: participant number, ‘-‘: prevaccination value excluded from the sample.

## Discussion

In contrast to glycoconjugate vaccines the immune response generated by polysaccharide vaccines is generally poorly immunogenic in infants, does not elicit a memory response, and produces antibodies of lower avidity with less bactericidal activity relative to IgG concentration [25]. Additionally, several polysaccharide vaccines including polysaccharide C [26] have been shown to elicit hyporesponsiveness, i.e. diminished immune response after booster compared to primary vaccination. The protective effect of meningococcal polysaccharide vaccines is strongly age dependent. Early clinical studies have shown inferior protection from invasive disease after polysaccharide A vaccination in children < 4 years [27], and no protection in recipients of polysaccharide C below 24 months of age [28]. While total antibody concentrations after polysaccharide C vaccination fails to explain higher susceptibility in immunized infants [29], the SBA assay, specifically a titer of ≥ 8 when measured with rabbit complement, is currently the most practical method for the prediction of immunity after vaccination [14]. It has superseded correlates based on antibody concentrations alone, e.g. an earlier proposal suggesting protection at concentrations of IgG ≥ 2.0 μg/ml [15].

While short-term qualitative and quantitative characteristics of the immune response to meningococcal polysaccharide vaccines have been extensively studied, less information is available on long-term effects. Antibody levels against serogroup C rapidly wane in infants [18,29] and seem to persist less than 4 years in children above the age of two [19]. Persistence of antibody levels in adults seems to last considerably longer, but very few studies have been published detailing long-term protection in older age groups: Zangwill et al. described elevated SBA titers to serogroup C (measured with rabbit complement) in adult vaccinees even after ten years [20].

In the present survey we have used a convenience sample of 20 individuals vaccinated only once with polysaccharide A, C, W135, and Y to analyze the association of observed antibody levels with time since immunization. Our analyses suggest that average durations of protective SBA titers to serogroups A, C, W135, and Y exceed 115 months. Previously published data for serogroup C may serve to validate the long-term predictive ability of our exponential model of decay: Zangwill et al. describe a rise of SBA titers from a pre-vaccination level of 13.6 to 1111.9 after vaccination. Using parameters 1111.9 and −0.032 (Table 2) as parameters L_0_ and k, respectively, our model predicts a SBA titer of 23.9 after 120 months (calculated as exp(log(1111.9) - 0.032 · 120)). This value is slightly below the observed value of 70 [20], which, assuming our model is valid, would correspond to a rate constant −0.023 (= log(70/1111.9)/120) and thus to a deviation of 28% regarding parameter k. Despite this small error, our model reproduces the original observation that titers after 10 years are still higher than pre-vaccination levels. We have found no further studies following up SBA levels exceeding five years after polysaccharide vaccination.

Moreover, we detailed the substantial inter-individual variability of both SBA titers and IgG levels directed against reference strains of targeted serogroups. The estimated minimal duration for W135 (0 months) suggests that some vaccinees may not attain lasting protection against this serogroup at all. Also, our model of decay for titers against W135 forecasts that a substantial part (approximately 23%) of vaccinees loses protection against this serogroup by 5 years, the time recommended for revaccination [10,13]. The lower level of protection against W135 compared to other serogroups was further suggested by the significantly lower proportion of protective titers (65%) in our sample. Also, the quantification of IgG revealed that GMC reacting with polysaccharide W135 was the lowest (2.1 μg/ml) among the serogroups analyzed. On the other hand, our decay models predict that minimal durations of protection against serogroups A, C, and Y appear to exceed 24 months and only minor proportions (≤ 5%) probably lose protection within 5 years. As absolute risk for acquiring meningococcal disease for laboratory workers is low [4], only few case reports exist that could corroborate or refute suggested minimal durations of protection. Of those, we found only one report that contained previous vaccination history with polysaccharide vaccine. It describes infection of a laboratory worker 67 months after application of meningococcal A + C polysaccharide vaccine (Sanofi Pasteur MSD) caused by a serogroup A meningococcal strain [9]. As our model forecasts a probability of > 5% of missing protection after this interval, it is plausible that the laboratory worker was not immune at the time of exposure.

In contrast to SBA titers, we could not detect a significant decay of IgG levels with time. This is most likely due to the small power of our analysis to detect decays with slopes barely differing from zero. Zangwill et al., using a sample of 40 adults, described a decrease of total anti-capsular antibody concentration against serogroups A and C 10 years after vaccination; nevertheless, they noted that it was smaller than that seen in SBA titers against serogroup C [20].

GMCs differed significantly across serogroups with GMC against serogroup A considerably higher (17.4 μg/ml) than that of other serogroups. It is unlikely, however, that this represents a vaccine effect, as unimmunized adults frequently show high baseline levels against this serogroup. Levels of IgG in unimmunized individuals were 1.5 μg/ml and even 17.5 μg/ml in a study encompassing unimmunized individuals from North America and Sudan, respectively [30]. The reasons for high pre-vaccination concentrations of IgG and other immunoglobulin classes remain unclear, yet it is likely that several commensal bacteria including *Escherichia coli*[31] and *Bacillus pumilus*[32] give rise to cross-reacting antibody populations. Their role in bactericidal immunity, however, seems to be minor [30]. Although we cannot confirm it for our sample given the lack of pre-vaccination samples, it is probable that observed high concentrations of IgG against serogroup A are a corollary of high pre-existing levels rather than exceptional immunogenicity of serogroup A polysaccharide within the administered vaccine.

Several authors have investigated the correlation of SBA titers and concentrations of IgG after meningococcal polysaccharide vaccination. Maslanka et al. found a positive correlation for all investigated age groups after vaccination with serogroup C polysaccharide, noting that correlation was lowest in 1 year olds with a correlation coefficient of 0.34 [29]. Granoff et al. observed increased correlation of high-avidity antibodies with SBA titers after serogroup C polysaccharide immunization and concluded that low-avidity, probably non-functional antibodies decrease correlation [33]. Also, moderate correlations between IgG and SBA titers of 0.56 and 0.37 were found in adults [34] and toddlers [35] after vaccination with serogroup A polysaccharide. Moreover, several groups reported no correlation between antibody concentrations and SBA titers against serogroup C in non-immunized individuals [33,36]. We found significant associations between antibody concentrations and SBA titers only against serogroups C and W135. For A and Y, however, it seems that antibody concentrations contribute little to the explanation of protection. While in the case of serogroup A this may be due to high and varying levels of cross-reactive antibodies, the reason for a missing association in serogroup Y remains elusive. In contrast to A the correlation coefficient in Y is so low that it seems unlikely that our failure to determine a positive association is due to low power of our sample.

Finally, we report the considerable proportion of incomplete protection (30%) in a convenience sample of laboratory workers. As recommended by several authorities including the RKI [12], polysaccharide-conjugate vaccine should be used to reinforce immunization and to avoid development of hyporesponsiveness. We have shown for a small subset, that, as expected, revaccination with conjugate vaccine is indeed effective in restoring SBA titers.

Our survey has several limitations. Firstly, our sample size is small with 20 individuals tested, which is due to the low availability of adult individuals who have been immunized once with meningococcal polysaccharide vaccine. This entails that our power to detect differences in the kinetics of antibody responses against different serogroups is small. Secondly, our sample is cross-sectional in nature, which leads to impaired temporal resolution in the description of decay. A consequence is that we are unable to test whether more elaborate models would provide a better fit to observed data; e.g. Zangwill et al. describe a decrease of titers by 94% in the first two years, which is considerably higher than a decrease of 54% predicted by our exponential model. Finally, we cannot say whether all individuals showed adequate response to the vaccine, as we do not have pre-vaccination samples. Judging from estimated L_0_ values, however, most participants will have attained SBA titres ≥ 8 after vaccination. In spite of these limitations, we have been able to correctly reproduce major long-term observations.

In summary, we present data on the long-term persistence of antibodies after meningococcal polysaccharide vaccination in adults, which represents an under-researched topic.

## Conclusions

Average duration of protection in adults immunized with meningococcal polysaccharide vaccine reaches ten years or more. Due to considerable inter-individual variability protection falls below the average estimate in a substantial proportion of adults immunized with polysaccharide formulations. Specifically, immunity against serogroup W135 may last less than five years, the period suggested by CDC [13] and UK’s Department of Health [10] for reimmunization, for approximately 23% of vaccinees. Thus, presented observations may contribute to reconsideration of the presently recommended periods for revaccination of adults at risk for occupational infection.

## Abbreviations

SBA: Serum bactericidal andibody; CDC: Centers of disease control and prevention USA; d.f: Degrees of freedom; DoH: Department of Health UK; GMC: Geometric mean concentration; GMT: Geometric mean titer; IgG: Immunoglobulin class G; RKI: Robert Koch Institute Germany

## Competing interests

The authors declare that they have no competing interests.

## Authors’ contributions

JE analyzed the data and drafted the manuscript. JF determined IgG concentrations and contributed to analysis and interpretation of data. RB determined IgG concentrations and contributed to analysis and interpretation of data. AT determined SBA titers and contributed to analysis and interpretation of data. MF contributed to analysis and interpretation of data. UV conceived the quality control survey, and contributed to analysis and interpretation of data. All authors critically revised the manuscript. All authors read and approved the final manuscript.

## Supplementary Material

Additional files 1: Table S1 Serum bactericidal antibody titers and concentration of IgG against meningococci of serogroups A, C, W135, and Y. PN: participant number, m: male, f: female, Months: months after vaccination corrected by probable time to achieve plateau antibody concentrations (10 days, see Methods), Age: age in years at vaccination, ‘-‘: value excluded from the sample. Click here for file
